# The “Stock of Time” Method: A New Approach to Calculate Indirect Costs and Benefits in Economic Evaluations

**DOI:** 10.1177/0272989X251333787

**Published:** 2025-04-25

**Authors:** Lucy Kok, Carl Koopmans

**Affiliations:** SEO Amsterdam Economics, University of Amsterdam, The Netherlands; SEO Amsterdam Economics, University of Amsterdam, The Netherlands; Vrije Universiteit Amsterdam, The Netherlands

**Keywords:** cost–benefit analysis, cost–utility analysis, health care, QALY, HRQoL score, leisure, production costs

## Abstract

**Background:**

Health interventions affect people’s welfare directly by impacting people’s health but also indirectly via a change in consumption and leisure time caused by the change in health. This study discusses 2 ongoing issues in the economic evaluation of health interventions. The first is how to value a change in the amount of leisure time of a patient. The second issue concerns the valuation of a change in production.

**Methods:**

We present a theoretical model that assumes that individual utility depends on health, consumption, and leisure time. We assume that the total stock of time consists of 3 components: leisure time, working time, and recovery time. The model takes a societal perspective and assumes that individuals optimize their utility, conditional on time and budget restrictions.

**Results:**

For the first issue, the model indicates that the value of a change in the stock of time available for leisure and work has to be added to the direct effects of a health intervention, instead of only a change in work hours. For the second issue, the model indicates that in case of a change in longevity, only the change in taxes paid may be counted because the income change is included in the value of the change in quality-adjusted life-years. A numerical example shows that this approach may counterbalance the potential overestimation of the welfare effects of treatments with the human capital method and underestimation related to the friction cost method.

**Conclusion:**

We propose a new method that includes the welfare effects of health interventions due to a change in the amount of leisure time and avoids double counting of welfare changes, which are included in the direct effects.

**Highlights:**

- We present a theoretical model and use it to analyze 2 issues in the economic evaluation of health interventions: the inclusion of leisure time and the valuation of production.
- The model indicates that the effects of health changes on the amount of both work and leisure hours need to be taken into account in economic evaluation.
- As to the valuation of production, the model indicates that in case of a change in longevity, only the change in taxes may be counted.
- We propose the “stock of time” method to value changes in working hours and leisure hours, which may counterbalance potential overestimates and underestimates in established methods.

Health interventions affect people’s welfare directly by improving health but also indirectly via a change in consumption and leisure time, caused by the change in health. The economic evaluation of health interventions from a societal perspective includes direct effects as well as indirect effects.

One of the basic issues in the literature concerning the indirect effects is the value of changes in leisure time and income of the patient due to a change in health. Should this be added to the direct health effects or not? The Washington Panel assumed that in assessing health-related quality of life (HRQoL), survey respondents will take into account their income level as well as their leisure time.^1^ However, many researchers nowadays agree that changes in income are not included in the HRQoL score and thus should be added separately. Current guidelines on health economics evaluations from a societal perspective recommend the inclusion of productivity changes.^2^ The issue of leisure time is still unresolved. Nyman^3^ argued that, as leisure is an element in health status questionnaires (such as the EQ-5D questionnaire) whereas income is not, only income should be added separately. Johannesson and Meltzer^4^ also assumed that the change in leisure time is captured by the HRQoL scores. The Second Panel on Cost-Effectiveness in Health and Medicine stated that evidence is not definitive that the effects of morbidity on leisure are necessarily reflected in the utility scores or quality-of-life weights.^5^ A second issue is about the valuation of production changes due to a change in health. In the literature, the human capital approach (HCA) and the friction cost approach (FCA) are used. The HCA assumes that the production loss caused by illness is the present value of all lost future production of the individual.^6^ In contrast, the FCA assumes that production falls only for a limited period, until the moment an unemployed worker replaces the absent worker.^7^

We provide a theoretical model that explicitly includes leisure time and consumer choices between consumption, work, and leisure to analyze these issues. The model takes a societal perspective and assumes that individuals optimize their utility, conditional on time and budget restrictions. It uses standard theory in labor economics on the tradeoff between work and leisure to calculate the willingness to pay for changes in consumption and leisure (see, for example, Varian^8^ or Borjas^9^). The model is suited to perform cost–utility analysis (CUA) and cost–benefit analysis (CBA). Both are tools for assessing the welfare effects of health interventions from a societal perspective; see Appendix 1 on the difference between these tools.

The model predicts the effects of health interventions or illness on the quality and quantity of consumption and leisure. We use a utility function that is multiplicative in health and the utility of consumption and leisure. This specification assumes that health enhances welfare by increasing utility from consumption and leisure. A change in the HRQoL score is interpreted as a change in the ability to enjoy consumption and leisure. As CBA and (to a lesser exent) CUA express costs and benefits in monetary terms, utility changes are translated in willingness to pay.

A crucial assumption in the model is that if someone gets ill, the total time available for work and leisure declines, leading to a new choice between work and leisure. That is, reduced working time does not automatically increase leisure time, as the total amount of time available for leisure and work might decrease as health deteriorates. This is because sick people might need more recovery time than healthy people do. They might need more sleep and time for personal care (such as medical treatment), which in the literature is defined as activities that are necessary for survival and that individuals have to perform for themselves.^10,11^ Podor and Halliday^12^ included “sleep,”“sleeplessness,”“personal grooming other than sleep,” and “health-related self care.”

The contribution of this article is the proposal of a new approach for the valuation of leisure time and changes in production, apart from the HCA and the FCA. The main differences from the former models are as follows:

- The assumption that the total stock of time is spent not only on work and leisure but also on recovery time.
- Utility is derived from the ability to enjoy consumption and leisure and from the amount of consumption and leisure. The ability to enjoy consumption and leisure is captured by the HRQoL score, whereas the amount of consumption and leisure is captured by the willingness to pay for a quality-adjusted life-year (QALY). Former models make no distinction between the ability to enjoy consumption and leisure and the quantity of consumption and leisure.

The model may serve as a basis for further discussion on the validity of the assumptions made. Being clear and explicit about assumptions and the underlying theory makes it possible to relax or change assumptions, to assess the impact on the results.

This article proceeds as follows. In the next section, we present the model. The third section discusses how the model sheds light on debates in the literature about the 2 issues mentioned above and presents numerical examples. The final section concludes.

## The Model

According to Bleichrodt and Quiggin^13^ and Hammitt,^14^ the equivalence of cost-effectiveness analysis and CBA requires that the utility function is multiplicative in health and the utility of consumption and leisure. We use the following utility function that expresses the lifetime utility of a person (U) as the present value of expected utility, which depends on health, wealth (consumption), leisure, and longevity:

(1) $$U=\sum_{t=0}^{D}h_{t}f(c_{t},l_{t})\rho^{-t}$$

where D is the time of death, h_t_ is the health state in period t, f(ct,lt) is the utility of consumption (c) and leisure (l) in period t, and ρ is the rate of time preference. We define HRQoL as the way the health state affects the utility of consumption and leisure.^15^ Following Hammit,^14^ the HRQoL in the context of our model is the response to a time-tradeoff (TTO) question in which the respondent states that a fraction h of lifespan D lived in full health is as desirable as lifespan D lived in health state h. The HRQoL is scaled so that a value of 1 corresponds to full health and 0 to a state of health as bad as dead. The HRQoL can be measured using the EQ-5d, for which the weights are based on a TTO value set.

As the term h_t_ enters multiplicatively in equation (1), we assume that health enhances the “quality” of life by increasing utility from consumption c_t_ and leisure time l_t_ (see Murphy and Topel^16^). Decreases in the utility derived from consumption and leisure due to decreases in health h_t_ stem from increased pain or discomfort. h_t_ can thus be interpreted as the ability to enjoy consumption and leisure.

Given this specification, the value of a QALY gained is the willingness to pay for 1 additional life-year in complete health. The value of a QALY therefore depends on the amount of consumption and leisure. The number of QALYs gained by interventions depends only on health and longevity, not on the amount of consumption and leisure. However, a change in health may change the amount of consumption and leisure and thus lead to a change of the value of a QALY. For example, an intervention may lead to a higher income, which may induce a higher willingness to pay for a life-year in optimal health.

Following Meltzer,^17^ we assume medical consumption (m)does not enter directly in the utility function, only via a change in health (which is already captured in h_t_). However, medical consumption does reduce other consumption. Throughout this article, we define consumption as nonmedical consumption. Medical consumption is treated separately. We assume further that consumption is equal to the number of working hours (e) multiplied by the gross hourly wage rate (w) minus taxes (i is the tax rate) plus nonlabor income (N) minus medical consumption (m). Nonlabor income not only includes benefits or allowances in case of sickness or unemployment but also reimbursement for medical costs by the government or from health insurance. This leads to the following budget constraint for consumers, where suffix t indicates the time period:

(2) $$c_{t}=e_{t}w_{t}\left(1-i_{t}\right)+N_{t}-m_{t}$$

The total amount of time available (T) consists of leisure time (l), working time (e), and recovery time (s) (e.g., sleep and personal care).

(3) $$T_{t}=l_{t}+e_{t}+s_{t}$$

In this, we follow Grossman^18^ who argued that “health can be viewed as a durable capital stock that produces an output of healthy time” and that “that a person’s . . . stock of health determines the total amount of time he can spend producing money earnings and commodities.” Thus, the healthier a person is, the smaller s and the larger the healthy time available for work and leisure. This is in accordance with the empirical analysis of the relation between health and time allocation, which shows that ill health leads to more hours of sleep and personal care.^11,12^

To calculate the societal welfare change, we add the external effects of the health change of patients on the rest of society (nonpatients). These consist of transfers to and from the patient in the form of paid taxes and received disability benefits, pensions, and health cost covered by public insurance.

Appendix 2 elaborates the model. According to the model, the social willingness to pay for indirect effects of a positive change in health and an increase in longevity on the amount of leisure time and working hours consist of 3 parts (selected parts from equation (27) in Appendix 2):

(4) $$\begin{array}{c} \sum_{t=0}^{D_{0}}\Delta \left[e_{t}w_{t}+l_{t}w_{t}\left(1-i_{t}\right)\right]\rho^{-t}+\\ \sum_{t=D_{0}+1}^{D_{1}}\Delta \left[e_{t}w_{t}+l_{t}w_{t}\left(1-i_{t}\right)\right]\rho^{-t}+\sum_{t=D_{0}+1}^{D_{1}}e_{t1}w_{t1}i_{t1}\rho^{-t} \end{array}$$

where D is life expectation, suffix 0 denotes the original longevity, and suffix 1 indicates the new longevity.

The first term in equation (4) reflects the change in the stock of time available for work and leisure due to a health intervention or disease during the original lifespan, where leisure is valued at the net wage rate w_t_(1 − i), while working hours are valued at the gross wage rate (w_t_). Time for recovery (s_t_) has a value in that it enhances health. It does not appear explicitly in equation (4) but implicitly as recovery time changes the time available for work or leisure. The productivity effect of a positive change in longevity consists of 2 parts:

1. The change in the stock of time available for work and leisure in the added life-years (the second term in equation (4)). This part is zero, unless there is a change in health in the added life-years compared with the health before treatment.
2. The taxes on labor income in the new health state in the added life-years (the third term in equation (4)). This term reflects the external effects on the rest of society of the added production in the added life-years.

The change in production is the value of the change in working hours. For simplicity, we restrict production to paid labor and exclude nonmarket production, such as household activities. Production must be distinguished from the change in consumption, as consumption also contains a change in transfers (nonlabor income and taxes). The change in transfers is, however, a redistribution item in CBA: a cost for society and a benefit for the patient, or the other way around. Transfers appear in the equation only for the welfare change due to a change in longevity because the counterpart of the change in transfers is contained in the value of a QALY (see Appendix 2, equations (27) and (28)).

In case of a negative or zero change in longevity ( $D_{0}\geq D_{1}$ ), equation (4) becomes (selected parts from equation (28) in Appendix 2):

(5) $$\sum_{t=0}^{D_{1}}\Delta \left[e_{t}w_{t}+l_{t}w_{t}\left(1-i_{t}\right)\right]\rho^{-t}-\sum_{t=D_{1}+1}^{D_{0}}e_{t0}w_{t0}i_{t0}\rho^{-t}$$

Note that the first term now reflects the change in the stock of time available for work and leisure during the new lifespan, as this is shorter than the original life span. The second term denotes the external effects of the production loss in the lost life-years on the rest of society. The second term in equation (4) is absent in equation (5), as there are no added life-years. The results of either equation (4) or (5) enter in the numerator of the cost–utility ratio (equation (1) in Appendix 1).

## Issues in CBA and CUA

How do the model results presented in the former section shed light on the issues described in the first section? In this section, we will elaborate the 2 issues: how to incorporate a change in leisure time and how to valuate extra production. We will also present a numerical example.

### Leisure Time

Our model assumes that changes in working time and in the quantity of leisure time are not included in the health state, while the quality of leisure time is included. Nyman^3^ stated that income effects are not included in the HRQoL measures but effects on leisure time are, because 3 of the 4 most widely used generic health status questionnaires include a question related to role functioning (EQ-5D, SF-6D, and QWB). For example, the EQ-5D questionnaire contains 5 dimensions: mobility, self-care, usual activities, pain/discomfort, and anxiety/depression. Respondents are asked to self-assess their functioning on each of these dimensions, on a 3-level scale. The ability to participate in paid or unpaid work is subsumed in the third dimension of the EQ-5D questionnaire, labeled “usual activities.” The 3 levels from which respondents choose on this dimension are:

- no problems with performing usual activities (e.g., work, study, housework, family or leisure activities),
- some problems with performing usual activities, and
- unable to perform usual activities.

According to Nyman (p. 1394):^3^

“By explicitly including these questions, the EQ-5D, SF-6D, and QWB capture the intrinsic value of the role functioning aspect of the health state, the aspect that relates most closely to the amount of leisure that the respondent would have in that health state.”

However, the question on role functioning has no references to the amount of leisure time nor to time for work. To the extent that respondents do indeed take into account the effects of their health condition on work and leisure, the EQ-5D questionnaire would seem to pick up the quality of leisure time and working time, not the quantity. Moreover, it would be inconsistent to assume that people do incorporate the quantity of leisure time in their HRQoL scores but not the quantity of working time, as the question is the same for working hours and leisure time. So, if the effects of a change in working hours are not included in the HRQoL score and have to be added separately (in terms of production loss), the same would hold for the effects of a change in leisure time.

There is a small body of literature that tries to establish if effects on income and leisure are included when assessing health states, based on questionnaires among health professionals^19^ and the general public.^20–22^ Respondents were asked to value 1 or more health states. These studies find that most respondents (ranging from 61% to 84%) said they spontaneously include effects on leisure time. The questionnaires do not distinguish between effects on the quality of leisure time and the quantity of leisure time. The studies find hardly any differences in HRQoL scores between respondents who did and did not consider leisure while valuing the health state. This seems logical: it is hard to imagine respondents would not spontaneously take the quality of leisure time into account as all 5 dimensions on the EQ-5D are relevant for the quality of leisure time (as impaired mobility, pain, and anxiety decrease the quality of leisure time). However, they found significantly lower HRQoL scores for respondents who thought the health state would affect their ability to enjoy leisure. This is consistent with our hypothesis that the quality of leisure is spontaneously included while rating HRQoL. The results of the scarce literature are thus consistent with the model in that the HRQoL scores of respondents take into account the ability to enjoy leisure (the quality of leisure time). The studies provide no evidence that the amount of leisure time is spontaneously included in the HRQoL score.

If the assumptions of the model are valid, the amount of leisure time has to be added separately to the direct health effects reflected in the QALY gain. Moreover, we propose that the change in leisure time is not measured as the inverse of the change in working hours. Surveys that measure the change in working hours due to a change in health thus should also measure the change in leisure time and recovery time. Empirical research shows that poor health is related to more recovery time and less time for paid work.^11,12^

To incorporate the total stock of time in health evaluations, 2 practical issues have to be tackled: what to include in recovery time and what is the value of leisure time? As to the definition of recovery time, Grossman^17^ distinguished time invested in health (such as preventive activities) and lost time due to sickness or injury. The definition in that empirical literature is in accordance with lost time due to sickness or injury, as this requires more sleep and personal care. Preventive activities, such as physical exercise or screening, are not distinguished in the empirical literature. Sports and fitness, for example, are defined as leisure time.^10,11^ Not only empirically, but also conceptually, it will be difficult to distinguish preventive activities from leisure time, as people may enjoy these activities. We therefore suggest considering these activities as leisure time. There is also discussion about including “eating and drinking at home.” Podor and Halliday^12^ saw this as leisure time, whereas Gimenez-Nadal and Molina^11^ viewed it as an activity to stay alive. Although eating and drinking are necessary for staying alive, the time spent on eating and drinking is a choice and might as well be counted as leisure time.

As to the valuation of leisure time, this is at the margin equal to the net wage rate (see Appendix 2). For nonmarginal changes, additional assumptions have to be made as the marginal value of leisure time diminishes as the amount of leisure time increases. It might be zero for someone who does not work. For the economic evaluation of nonmarginal changes of leisure time, the average value of leisure time gained or lost has to be taken into account, not the marginal value. This value will be higher than 0% and lower than 100% of the net wage. A study of Verbooy et al.^23^ finds a willingness to pay for leisure time of €12.27 per hour for people with paid employment (leaving €0 responses out) based on a contingent valuation study in the Netherlands in 2018. As the average net wage rate in 2018 was about €18 per hour (a gross wage rate of €22 per hour), the value of leisure time would have been roughly 70% of the average net wage rate. We recommend performing sensitivity analysis with different percentages of the net wage.

### Valuation of Changed Production

Most guidelines for health evaluations do not take into account productivity changes due to health changes.^2^ Guidelines that take a societal perspective do incorporate productivity changes.^2^ However, there is an ongoing debate about the method to value such changes in production due to health changes. Both the HCA and the FCA are proposed, with relatively few studies using the latter method.^24^

The HCA assumes that the value of the production change is the present value of all lost or gained future production of the individual.^6^ Guidelines recommend using the average gross wage to calculate the value of production changes.^2^ In case of a change in longevity, there has been discussion about double counting, as the consumption in lost or gained life-years is already captured by the value of a QALY,^25,26^ which is consistent with our model. The “net human capital approach,” as Johannesson^26^ called it, has not become standard practice in health evaluations, although the Danish guideline proposes to perform a sensitivity analysis with this approach.^27^

The FCA takes a short-run perspective. In the short run, there might be unemployment, and workers can be replaced by unemployed persons. However, business cycles with high and low unemployment alternate. The FCA would curiously imply that there is no productivity gain of getting sick people—who have already been replaced—back to work, even if recovery occurs in a tight labor market. Moreover, if a sick worker can be replaced by an unemployed person, it would not be costless, because the unemployed person taking over the work of the sick person would lose leisure time.^28^ Friction cost proponents indicated that leisure is not treated as “costless” but rather that gains and losses of leisure at a societal level even out between the sick worker and the replacing worker.^29,30^ They thus implicitly assume that health does not influence recovery time.

### Numeric Examples

The following numeric examples indicate that the results of our model —the stock of time approach (STA)—will often yield results that are in between the HCA and the FCA. The example is inspired by an economic evaluation of the costs of head and neck cancer, which calculates the production losses of the cancer using the HCA and the FCA.^31^ We simplified the underlying data to give a clearer insight in the consequences of the methods and added possible effects of a hypothetical intervention to cure this disease.

Individuals are assumed to be diagnosed with the cancer at an age of 50 y. Because of the illness, 20% of the patients die at an age of 51 y. The amount of time available for work and leisure before the illness was 16 h per day: 24 h minus 8 h of recovery time (5,840 h per year). Because of the illness, survivors decrease working hours by, on average, 2 h per day (730 h per year). To illustrate the effect of recovery time, we consider 2 situations. In situation 1, the average amount of recovery time stays the same, which means leisure time increases with 730 h a year, as working time decreases with 730 h. In situation 2, recovery time increases with 4 h per day—1,460 h per year. This means the amount of leisure time decreases with 730 h per year.

Table 1 presents the total welfare loss of the illness due to a change in working hours and leisure time, calculated using the 3 methods: the HCA, FCA, and STA. The calculations assume that premature death leads to a loss of 14 productive years (from age 51 y to retirement age at 65 y). We assume a gross wage rate of €20 per hour and an average tax rate of 20%. Furthermore, for the FCA, a friction period of 16 wk is assumed. For simplicity, a discount factor of 0% is used. The average willingness to pay for leisure time is assumed to be 70% of the net wage. Table 2 also contains the direct welfare losses in terms of lost QALYs, which are valued at €300,000 per patient.

**Table 1:** Average Welfare Loss per Patient Calculated with the HCA, FCA, and STA

Welfare losses	HCA	FCA	STA, Situation 1	STA, Situation 2
1. Value of lost production due to premature death (20% of patients)	−€102,200	−€2.246	−€20.440	−€20.440
2. Value of lost production per survivor	−€163,520	−€3.594	−€163.520	−€163.520
3. Value of lost/gained leisure time survivors	—	€0	€91.571	−€91.571
Total welfare loss production and leisure time	−€265.720	−€7.600	−€92.389	−€275.531
Value lost QALYs	−€300,000	−€300.000	−€300.000	−€300.000
Total welfare loss	−€565,720	−€307,600	−€392.389	−€555.091

HCA, human capital approach; FCA, friction cost approach; QALY, quality-adjusted life-year; STA, stock of time approach.

Change in production in HCA and STA cumulated over a period of 14 y. Change in production in FCA cumulated over a period of 16 wk.

**Table 2:** Average Costs and Benefits per Patient Calculated with the HCA, FCA, and STA

Costs and benefits	HCA	FCA	STA, Situation 1	STA, Situation 2
Benefits (10% of welfare loss)	€56,572	€30,760	€39,239	€57,553
Cost of the intervention	−€50,000	−€50,000	−€50,000	−€50,000
Net benefits	€6,572	−€19,240	−€10,761	€7,553

HCA, human capital approach; FCA, friction cost approach; STA, stock of time approach.

As Table 1 indicates, the largest welfare loss due to premature death occurs in the HCA. This is because all lost production is valued. The FCA leads to far lower costs because only 16 wk of production loss are counted, instead of 14 y. According to the STA, the production loss due to premature death is only the external effect on the rest of society, which is 20% (the tax rate) of the production loss in the HCA calculation. The HCA thus leads to a large overestimation of the welfare loss due to premature death, while the FCA leads to an underestimation given a friction period of 16 wk. The value of lost production of survivors amounts to €163.520 in the HCA and the STA. According to the FCA, this welfare loss is only €3.594, as it is calculated over the friction of 16 wk, instead of over 14 y. The gain or loss in leisure time of survivors is not taken into account in the HCA, while the FCA assumes the value of gained leisure time is zero, because it evens out with the lost leisure time of the unemployed who take over the paid work after the friction cost period.

In situation 1, the leisure time for the patient increases, which decreases the total welfare loss due to the lost working hours. The STA calculations value the increased leisure time of the patient at 70% of the net wage rate, so, in total, at 56% of the gross wage (70% × 80%). Compared with the HCA, the STA leads to a lower total welfare loss by taking into account the gain in the leisure time of the patient. As compared with the FCA, the STA leads to a higher total welfare loss because the value of lost production according to the FCA is measured over a period of 16 wk, instead of 14 y.

In situation 2, the patient loses leisure time, leading to a larger welfare loss than in situation 1 according to the STA. The STA result now is almost equal to the HCA. The reasoning behind this result is, however, quite different. On one hand, the value of lost production due to premature death in the STA is smaller than in the HCA; on the other hand, the HCA does not take the value of lost leisure time of survivors into account.

A health intervention to cure or prevent the illness will have higher benefits in case of larger welfare losses due to the illness. Suppose the intervention would decrease the welfare losses by 10% and the cost of the intervention is €50,000. Table 2 shows that this health intervention would lead to estimated welfare gains if the indirect welfare effects are calculated with the HCA and with the STA, but only in case illness leads to a decrease in leisure time (situation 2). Using the FCA will lead to estimated negative welfare gains, as will the STA if the illness does lead to a gain in leisure time.

The overestimation and underestimation of the HCA and the FCA depend heavily on the nature of the illness or intervention. The HCA’s overestimation will be especially high if there is a significant change in longevity. The underestimation of the FCA will be particularly large in cases of longer periods of sickness and a loss of leisure time due to sickness.

## Conclusion and Discussion

The model leads us to conclusions on 2 ongoing issues in CBA. The first issue concerns the valuation of a change in leisure time of the patient due to a change in health. Productivity changes due to a change in working hours are generally included in CBAs and CUAs from a societal perspective, but leisure time is not. We suggest adding a change in the total amount of time available for leisure and work to the direct health effects. At the margin, the value of an extra hour of leisure time is equal to the net wage rate, whereas an extra hour of working time is valued at the gross wage rate.

A second issue is the valuation of production changes due to a change in health using the HCA or the FCA. The model indicates that the FCA may underestimate welfare losses due to ill health because it implicitly assumes that the total stock of time available for work and leisure is not affected by health. On the other hand, the HCA may overstate production losses of lost life-years, as it estimates the loss of working hours until death valued at the gross wage. According to the model, only the taxes forgone should be counted because the lost working hours valued at the net wage rate are included in the value of the lost QALYs. Moreover, in case of morbidity, the HCA may overestimate the welfare effect if patients gain leisure time through treatments, which the HCA ignores.

Incorporating the outcomes of the model in CBA and CUA will also have redistribution effects compared with the HCA and the FCA. The STA might favor interventions for people without a paid job (such as retirees and unemployed individuals), as for them better health will lead to more leisure time, which is not included in the HCA and the FCA. The STA might favor interventions for the elderly compared with the HCA because the production loss of people dying young has less weight in the STA, as it values the effect of changes in longevity on productivity by only the taxes forgone. However, the STA might favor interventions for the young compared with the FCA, because external effects over all lost life-years are taken into account, instead of only the production losses in the friction period.

Our model clarifies the basic relations between consumption, leisure, and health. The model has a simplified nature: we assume a multiplicative utility function, the time of death is known, and there is a proportional tax rate. The model does not include savings, but it does include transfers from the rest of society. As in most Western countries, health expenses are (partly) paid out of health insurance or taxes. These transfers have to some extent the same effect as allowing savings: both allow income smoothing over the life cycle. A crucial assumption in the model is that the total amount of time available for work and leisure is dependent on health. Spending more time on sleeping and other health-related activities, such as hospital stays, reduces the total stock of time. The analysis can be extended by relaxing some assumptions. For example, the time spent on housekeeping activities and informal care can be added as these can be substantial.^32^ Within the context of the current article, however, our aim is to show how health and longevity changes may create welfare changes through labor and leisure changes, even in a simple model. Further work is needed to assess the effects in more complicated models. Furthermore, we calculated only the willingness to pay for marginal utility changes caused by a change in leisure time. For nonmarginal changes in leisure time, additional assumptions have to be made. These additional refinements will, however, not change the main conclusions.

## Supplemental Material

sj-docx-1-mdm-10.1177_0272989X251333787 – Supplemental material for The “Stock of Time” Method: A New Approach to Calculate Indirect Costs and Benefits in Economic Evaluations Supplemental material, sj-docx-1-mdm-10.1177_0272989X251333787 for The “Stock of Time” Method: A New Approach to Calculate Indirect Costs and Benefits in Economic Evaluations by Lucy Kok and Carl Koopmans in Medical Decision Making

sj-docx-2-mdm-10.1177_0272989X251333787 – Supplemental material for The “Stock of Time” Method: A New Approach to Calculate Indirect Costs and Benefits in Economic Evaluations Supplemental material, sj-docx-2-mdm-10.1177_0272989X251333787 for The “Stock of Time” Method: A New Approach to Calculate Indirect Costs and Benefits in Economic Evaluations by Lucy Kok and Carl Koopmans in Medical Decision Making
