# Supplementary material for: The “Stock of Time” Method: A New Approach to Calculate Indirect Costs and Benefits in Economic Evaluations
Source: Med Decis Making. 2025 Apr 25;45(5):614–22. doi: 10.1177/0272989X251333787 (PMC12166135; doi:10.1177/0272989X251333787)
Supplement: sj-docx-1-mdm-10.1177_0272989X251333787 – Supplemental material for The “Stock of Time” Method: A New Approach to Calculate Indirect Costs and Benefits in Economic Evaluations [file sj-docx-1-mdm-10.1177_0272989X251333787.docx]

### Appendix 1 Cost-utility analysis and cost-benefit analysis

Cost-benefit analysis (CBA) and cost-utility analysis (CUA) are tools to evaluate health interventions. With these tools the costs of an intervention are weighed against the benefits, to assess if it is welfare enhancing (CBA) or how the health benefits in terms of QALY’s (Quality Adjusted Life Years) relate to the extra costs (CUA).^1^ A QALY is the product of the number of life-years affected by an intervention or disease and the health related quality of a life year (HRQoL). We define HRQoL as the way the health state affects the quality of life.^2^ In the context of our model, the HRQoL is the response to a time-trade-off (TTO) question in which the respondent states that a fraction of lifespan X lived in full health is as desirable as lifespan X lived in health state h_t_, see Hammit ^3^. It is expressed in a figure from 0 to 1, where 0 is regarded as equal to death and 1 is regarded as perfect health. The EQ5D is an often used questionnaire to measure the HRQoL. It contains five questions about the health status. The scores on these questions are translated in a number from 0 to 1 by weighing the scores on each question with a preference based weight that is based on time-trade-off (TTO) questions comparing different health status or how much risk with their life they are willing to take to

avoid ill health (standard gamble).^2^

Cost-Utility Analysis (CUA) is a Cost Effectiveness Analysis (CEA) that involves the most general health care outcome, which is a Quality Adjusted Life Year (QALY)^1^. It is a special case of a CEA, which results can also be expressed in terms of e.g. blood pressure, infections or mortality rates. Like CEA, CUA results are usually expressed in terms of a ratio. The numerator of this ratio includes the direct financial costs of the intervention and - in case of a societal perspective - the indirect costs to the patient and to society. The denominator reflects a gain in health from an intervention. The cost-utility ratio thus measures the additional costs per QALY gained. The intervention is regarded beneficial from a societal perspective if the cost-utility ratio does not exceed a previously set value of a QALY. This can be written as:

$$\frac{\Delta costs}{\Delta QALY}<V_{MAX} (1)$$

Where $\Delta QALY$ is the number of QALY’s gained and $V_{MAX}$ is the threshold value for a QALY gained. Δcosts reflects cost changes, which consist of the change in medical costs due to the intervention and may also include any further savings or additional costs related to changes in productivity, informal care and patient-related costs like travel costs.

Cost-benefit analysis (CBA) assesses if the welfare gains of the interventions are larger than the costs. In that case the welfare gain is larger than zero. All costs and benefits are expressed in monetary terms. This condition can be written as:

$\Delta QALY*V_{QALY}-\Delta costs>0 (2)$

The first term is the direct health benefit: the number of QALY’s gained multiplied by the monetary value of a QALY gained. As both CBA and CUA are based on utility changes, the value of a QALY is derived from the willingness to pay for a QALY, not from the marginal costs of producing a QALY. The marginal costs of producing a QALY are more appropriate to be used in health evaluations from a healthcare payer’s perspective, see Claxton et al.(2015).

It is easy to see that equation 1 can be rewritten as equation 2 if we assume that the threshold value $V_{MAX}$ is equal to $V_{QALY}$. Cost-benefit analysis and cost-utility analysis are thus essentially the same, if they are conducted from a societal perspective.

References

1. Brent, R. J.. Cost-benefit analysis versus cost-effectiveness analysis from a societal perspective in Healthcare. *International Journal of Environmental Research and Public Health*, 2023; *20*(5): 4637. doi: 10.3390/ijerph20054637
2. Karimi, M., & Brazier, J. Health, health-related quality of life, and quality of life: what is the difference?. *Pharmacoeconomics*, 2016; *34*: 645-649. doi: 10.1007/s40273-016-0389-9
3. Hammitt JK. Admissible utility functions for health, longevity, and wealth: Integrating monetary and life-year measures. *J Risk Uncertain*. 2013;47(3):311-325. doi:10.1007/s11166-013-9178-4
