# Supplementary material for: The “Stock of Time” Method: A New Approach to Calculate Indirect Costs and Benefits in Economic Evaluations
Source: Med Decis Making. 2025 Apr 25;45(5):614–22. doi: 10.1177/0272989X251333787 (PMC12166135; doi:10.1177/0272989X251333787)
Supplement: sj-docx-2-mdm-10.1177_0272989X251333787 – Supplemental material for The “Stock of Time” Method: A New Approach to Calculate Indirect Costs and Benefits in Economic Evaluations [file sj-docx-2-mdm-10.1177_0272989X251333787.docx]

Appendix 2 The model

This appendix elaborates the model. It is divided in three sections:

1. Welfare changes for patients
2. Welfare change for the rest of society
3. Total welfare change

The basic model has been introduced in section 2 of the paper. It consists of a utility function which is multiplicative in health and the utility of consumption and leisure. We use the following utility function which expresses the lifetime utility of a representative person as the present value of expected utility which depends on health, wealth (consumption), leisure and longevity:

$$U=\sum_{t=0}^{D} h_{t}f(c_{t},l_{t})\rho^{-t} (1)$$

where D is the time of death, h_t_ is the health state (HRQoL) in period t, f(c_t_,l_t_) is the utility of consumption (c) and leisure (l) in period t and ρ is the rate of time preference.

Utility is increasing in consumption and leisure, showing diminishing returns: ${\partial f}/{\partial c}>0$, ${\partial f}/{\partial l}>0$, ${\partial^{2}f}/{\partial c^{2}<0}$, ${\partial^{2}f}/{\partial l^{2}<0}$. Furthermore, consumption and leisure are complements: the marginal utility of leisure increases if consumption increases, and the other way around: ${\partial^{2}f}/{\partial c\partial l>0}$. That is, leisure time yields more utility when combined with consumption in the form of leisure time activities, like going to a restaurant or to the movies. If we add a budget restriction in which consumption depends on working (via income), consumption and leisure are substitutes: consumption is paid for by earning an income and thus giving up leisure time, unless there is non-labour income. The health state h_t_ is a measure which ranges from 0 to 1, where 1 corresponds to optimal health and 0 corresponds to a health state judged equivalent to death.

The budget constraint for consumers is:

$c_{t}=e_{t}w_{t}\left( 1-i_{t} \right)+N_{t}-m_{t}$ (2)

Where w_t_ is the gross hourly wage rate in period t, e_t_ is the time spent working in period t, i_t_ is the tax rate in period t, N_t_ is non-labour income in period t and m_t_ are the medical costs in period t. Medical costs depend on health, so m_t_=g(h_t_). For simplicity, savings and loans are ignored. However, transfers have to some extent the same effect as allowing savings and loans: both allow income smoothing over the life cycle.

The total amount of time available (T) consists of leisure (l), working time and time for sleep and personal care (s). Time for sleep and personal care depends on health, so s_t_=j(h_t_).

$$T_{t}=l_{t}+e_{t}+s_{t} (3)$$

We do not explicitly solve the utility maximisation problem. In the next sections, we will derive conclusions directly from equations (1)-(3).

### Welfare changes for patients

Now consider in more detail the change in utility which would result from a change in time of death from D_0_ to D_1_ due to an intervention or disease, a change in health state from h_t0_ to h_t1_, or a change in the utility derived from consumption and leisure:

$$U_{1}-U_{0}=\sum_{t=0}^{D_{1}} h_{t1}f(c_{t1},l_{t1})\rho^{-t}-\sum_{t=0}^{D_{0}} h_{t0}f(c_{t0},l_{t0})\rho^{-t} (4)$$

For positive changes in longevity (when D_1_ >D_0_), decomposing the first term in equation (4) in non-added (t=0 🡪 D_0_) and added life years ( t=D_0+1_ 🡪D_1_). yields:

$$U_{1}-U_{0}=\sum_{t=0}^{D_{0}} h_{t1}f(c_{t1},l_{t1})\rho^{-t}+\sum_{t=D_{0}+1}^{D_{1}} h_{t1}f(c_{t1},l_{t1})\rho^{-t}-\sum_{t=0}^{D_{0}} h_{t0}f(c_{t0},l_{t0})\rho^{-t} (5)$$

As h_t1_- h_t0_=∆h_t_ we can replace h_t0_ in the third term of equation (5) by h_t1_-∆h_t_ yielding:

$$U_{1}-U_{0}=\sum_{t=0}^{D_{0}} h_{t1}f(c_{t1},l_{t1})\rho^{-t}+\sum_{t=D_{0}+1}^{D_{1}} h_{t1}f(c_{t1},l_{t1})\rho^{-t}-\sum_{t=0}^{D_{0}} {(h}_{t1}{-\Delta h}_{t})f(c_{t0},l_{t0})\rho^{-t} (6)$$

The last term in equation (6) can now be split in two terms, of which one can be added to the first term in equation (6) yielding:

$$U_{1}-U_{0}=\sum_{t=0}^{D_{0}} h_{t1}[f(c_{t1},l_{t1})-f(c_{t0},l_{t0})] \rho^{-t}+\sum_{t=D_{0}+1}^{D_{1}} h_{t1}f(c_{t1},l_{t1})\rho^{-t}-\sum_{t=0}^{D_{0}} {-\Delta h}_{t}f(c_{t0},l_{t0})\rho^{-t} (7)$$

Bringing the third term of equation (7) to the front and replacing (f(c_t1_, l_t1_)- f(c_t0_, l_t0_) in the first term of equation (7) by ∆f(c_t_,l_t_) yields:

$$U_{1}-U_{0}=\sum_{t=0}^{D_{0}} {\Delta h}_{t}f(c_{t0},l_{t0})\rho^{-t}+\sum_{t=0}^{D_{0}} h_{t1}\Delta f(c_{t},l_{t})\rho^{-t}+\sum_{t=D_{0}+1}^{D_{1}} h_{t1}f(c_{t1},l_{t1})\rho^{-t} (8)$$

For negative changes in longevity (when D_0_ >D_1_) the utility change can be decomposed as:

$$U_{1}-U_{0}=\sum_{t=0}^{D_{1}} {\Delta h}_{t}f(c_{t0},l_{t0})\rho^{-t}+\sum_{t=0}^{D_{1}} h_{t1}\Delta f\left( c_{t},l_{t} \right)\rho^{-t}-\sum_{t=D_{1}+1}^{D_{0}} h_{t0}f(c_{t0},l_{t0})\rho^{-t} (9)$$

Equations (8) and (9) show that the utility change for the patient consists of three parts. The first part reflects the utility change in the original life span due to a change in the HRQoL, which indicates the effect of the health state (${\Delta h}_{t})$on the ***quality* of consumption and leisure** . The second part reflects the utility change in the original life span due to a change in the ***quantity* of consumption and leisure**. The last part of the equations reflects the welfare effect of a change in **longevity** from D_0_ to D_1_. We will elaborate these three parts of the welfare change subsequently.

**Quality of consumption and leisure**

It is common practice in cost-benefit analysis (CBA) to calculate the willingness to pay for the utility change in the first component in equation (8) and (9) as the product of the change in health (in terms of QALYs gained or lost) and the value of a QALY. The value of a QALY is the willingness to pay (WTP) per additional life year in complete health. We now compute the marginal WTP in the context of our model, in two steps. First, we look at changes in health ($dh_{t}$) and consumption ($dc_{t}$), holding utility constant and using derivatives of the utility function (equation (1)). These changes satisfy this equation:

$$\Delta U=0= \frac{\partial U}{\partial h_{t}}dh_{t}+\frac{\partial U}{\partial c_{t}}dc_{t}= \frac{\partial h_{t}f\left( c_{t0},l_{t0} \right)}{\partial h_{t}}dh_{t}+\frac{\partial h_{t}f\left( c_{t},l_{t} \right)}{\partial c_{t}}dc_{t} (10)$$

where $dh_{t}$ is positive and $dc_{t}$ is negative (i.e., a loss of consumption through a payment).

In the second step we derive the marginal rate of substitution between consumption and health from equation (10). This marginal rate of substitution is the amount of consumption that a consumer is willing to give up in exchange for an increase in health, while maintaining the same level of utility. It is thus the (positive) willingness to pay for an increase in health.

$$\mathrm{WTP}_{\mathrm{QALY}}=\frac{-dc_{t}}{dh_{t}}=\frac{\frac{\partial h_{t}f\left( c_{t0},l_{t0} \right)}{\partial h_{t}}}{\frac{\partial h_{t}f\left( c_{t},l_{t} \right)}{\partial c_{t}}}=\frac{f\left( c_{t0},l_{t0} \right)}{h_{t}f_{c}\left( c_{t},l_{t} \right)} (11)$$

Where:

$$f_{c}\left( c_{t},l_{t} \right)=\frac{\partial f\left( c_{t},l_{t} \right)}{\partial c_{t}}$$

Equation (11) shows that the value of a QALY differs between persons. It increases in consumption and leisure in period t_0_ and decreases in health. Hammitt^3^ reaches the same conclusion, although his utility function is somewhat different. He states that as the marginal willingness to pay decreases with health, using a fixed monetary value per life year is inconsistent with individuals’ preferences. However, for ethical reasons cost-benefit analyses researchers use one value for all persons, independent of health or wealth. It is not considered as ethical to value a life year in perfect health differently for poor and rich people, or for healthy and sick people. Accordingly, the value of a QALY (V_QALY_) can be interpreted as the average willingness to pay for a change in $f(c_{t0},l_{t0})$ for the whole population. Thus:

$$V_{\mathrm{QALY}}=\frac{1}{S}\sum_{i=1}^{S} {\mathrm{WTP}_{\mathrm{QALY}}}_{i} (12)$$

where S denotes the size of the population. The first component in equation (8) is the utility change caused by a change in the quality of consumption and leisure:

$$\Delta U_{quality consumption and leisure}=\sum_{t=0}^{D_{0}} {\Delta h}_{t}f(c_{t0},l_{t0})\rho^{-t} (13)$$

We now replace the utility change in equation 13 by the willingness to pay for this utility change. To do so, we replace the utility of one year in good health (${f(c}_{t0},l_{t0})$) with the willingness to pay for one year in good health, which is on average V_QALY_ according to equation 12:

$${WTP}\left( \Delta U_{quality consumption and leisure} \right)= \sum_{t=0}^{D_{0}} {\Delta h}_{t}V_{\mathrm{QALY}}\rho^{-t} (14)$$

This is the direct welfare change included in standard CBA.

In case of a loss of life years the social willingness to pay for the first component in equation (9) can be approximated by:

$${WTP}_{quality consumption and leisure,D_{0}>D_{1}}=\sum_{t=0}^{D_{1}} {\Delta h}_{t}V_{\mathrm{QALY}}\rho^{-t} (15)$$

**Quantity of consumption and leisure**

The second component of equations (8) and (9) expresses the change in utility due to a change in the amount of consumption and leisure. Rewriting consumption in terms of wage and non-wage income and medical costs, the second component of equation (8) becomes:

$$\Delta U_{quantity consumption and leisure}=\sum_{t=0}^{D_{0}} h_{t1}\Delta f(c_{t},l_{t})\rho^{-t}=\sum_{t=0}^{D_{0}} h_{t1}\Delta f[e_{t}w_{t}\left( 1-i_{t} \right)+N_{t}-m_{t} ,l_{t}]\rho^{-t} (16)$$

To assess the utility change caused by a change in the quantity of consumption and leisure we use a standard microeconomic framework, see for example Varian^1^ or Borjas^2^. In this framework, individuals seek to maximize their well-being by consuming goods and leisure. The assumption is that they are free to choose their working hours, thus there is (in the long run) no involuntary unemployment. If non-labour income is absent or low, individuals choose to work in order to earn money to buy goods. So there is a trade-off between consumption and leisure. Different combinations of consumption and leisure might yield the same level of utility. The choice between consumption and leisure is made within the budget constraint and the time constraint, equations (2) and (3), respectively.

Figure 1 illustrates the labour-leisure choice. Panel a shows that the total amount of time available for work and leisure is l_H_+e_H_ in a healthy state. The budget line for a healthy person (the upper red line) is determined by l_H_+e_H_ and the hourly net wage rate (in this example we assume that medical costs are zero, for simplicity). If a person does not work (e_H_=0) and there is no non-labour income (an assumption in this example, not in our model), total income and thus consumption are zero. If, on the other hand, the person has no leisure time and spends all available time on work (l_H_=0) consumption amounts to c-max_H_. Different combinations of consumption and leisure which yield the same level of utility in a healthy state are shown by the indifference curve U_H_. Utility is maximized by an optimal combination of leisure l_H_ (implying working hours of e_H_) and consumption c_H_. This is where the budget line touches the indifference curve U_H_. As health deteriorates, the total stock of time available for work and leisure (l_H_+e_H_) decreases as the time for sleep and treatment increases from s_H_ to s_S_. If the hourly wage rate stays the same (which we assume in this example, not in our model), the budget line shifts down parallel to the original budget line. This makes the highest attainable indifference curve the curve to U_S_. The optimal choice in this example is to decrease leisure time from l_H_ to l_S_ and decrease working hours from e_H_ to e_S_. This leads to a lower consumption level (c_S_) and less leisure (l_S_) than in the healthy state. In panel a no sickness allowance or other non-labour income is granted. If an allowance is present (N >0), as shown in panel b of figure 1, people might reduce their working hours in order to increase the amount of leisure. A sickness allowance (N_S_) shifts the budget line up, which will lead to a higher utility level in the sick state. In that case the utility derived from the amount of consumption and leisure might also be the same or even higher than in the healthy case.

Figure 1 The trade-off between consumption and leisure


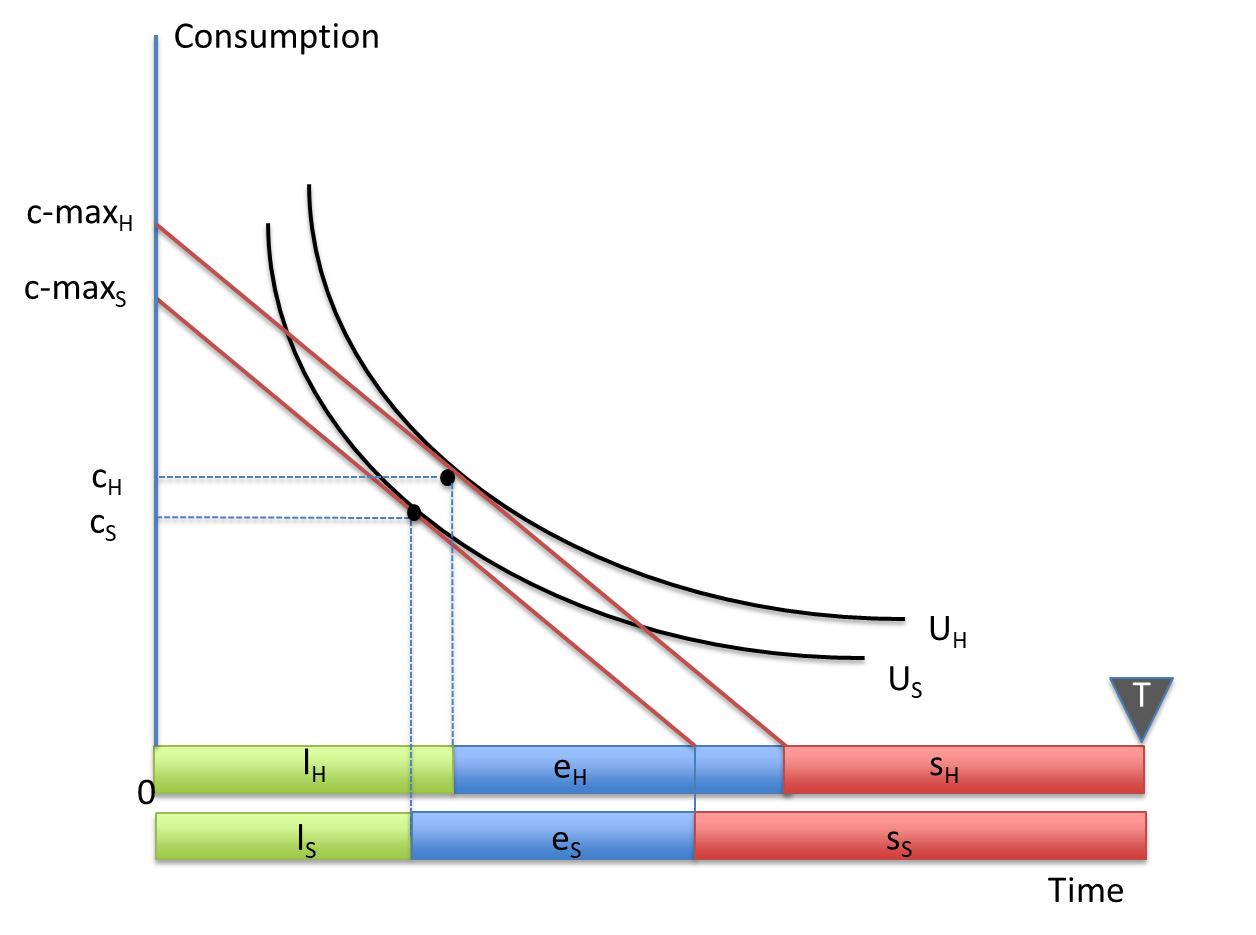

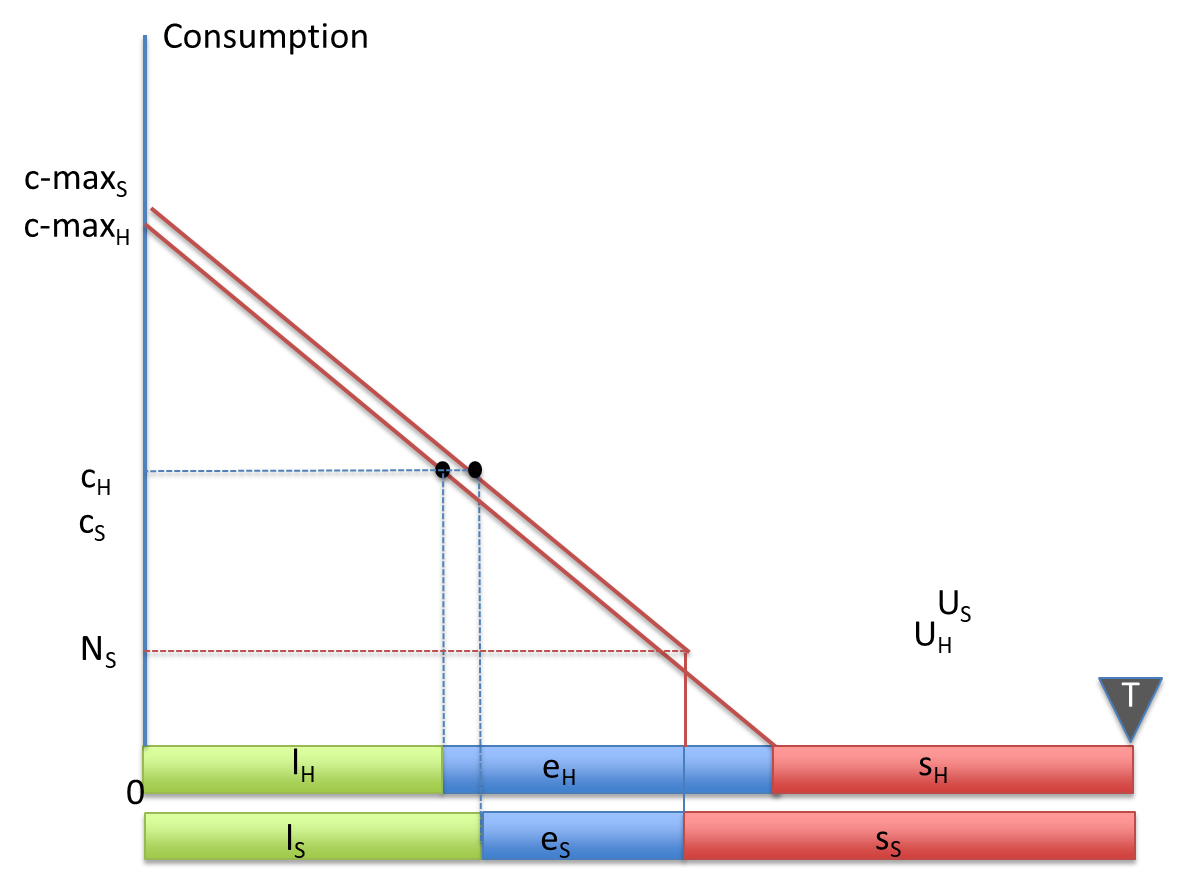


Panel a

Panel b

The utility change resulting from a change in leisure and consumption is:

${dU_{quantity consumption and leisure}=dl}_{t}\frac{\delta{(h}_{t}f\left( c_{t},l_{t} \right))}{\delta l_{t}}+\mathrm{dc}_{t}\frac{\delta(h_{t}f\left( c_{t},l_{t} \right))}{\delta c_{t}}=h_{t}\left[ \mathrm{dl}_{t}\frac{\delta f\left( c_{t},l_{t} \right)}{\delta l_{t}}+\mathrm{dc}_{t}\frac{\delta f(c_{t},l_{t})}{\delta c_{t}} \right] (17)$

The slope of the indifference curve dc_t_/dl_t_equals the marginal rate of substitution (MRS) in consumption. It represents the individual’s willingness to trade consumption for additional leisure time. Keeping utility constant (dU=0) and rearranging equation (17) yields:

$$\frac{\mathrm{dc}_{t}}{\mathrm{dl}_{t}}=-\frac{\frac{\delta f\left( c_{t},l_{t} \right)}{\delta l_{t}}}{\frac{\delta f\left( c_{t},l_{t} \right)}{\delta c_{t}}} (18)$$

Utility is maximised if the slope of the indifference curve is equal to the slope of the budget constraint. The slope of the budget line is the net wage rate. Thus, at the optimum, the marginal rate of substitution between leisure and consumption in equation (18) equals the net wage rate $w_{t}\left( 1-i_{t} \right)$.

We now assess the willingness to pay for the utility changes in equation (16), given what we know about the willingness to pay for leisure based on equations (17) and (18). By definition, the willingness to pay for a change in the budget for consumption equals 1. As the marginal rate of substitution between leisure and consumption is $w_{t}\left( 1-i_{t} \right)$, the willingness to pay for a change in leisure time can be approximated by$\Delta l_{t}w_{t}\left( 1-i_{t} \right).$ Medical costs substitute non-medical consumption and thus reduce utility from non-medical consumption. The value of this decline is equal to the costs of the intervention, which is the change in medical costs due to the illness. The willingness to pay for a change in the amount of consumption and leisure can thus be approximated by:

$$\mathrm{WTP}_{consumption and leisure}=\sum_{t=0}^{D_{0}} \Delta[e_{t}w_{t}\left( 1-i_{t} \right)+N_{t}-m_{t}+ l_{t}w_{t}\left( 1-i_{t} \right)]\rho^{-t} (19)$$

Rewriting yields:

$$\mathrm{WTP}_{consumption and leisure}=\sum_{t=0}^{D_{0}} \left[ {\Delta[(e_{t}+l}_{t}{)w}_{t}(1-i_{t})]+{\Delta N}_{t} \right]\rho^{-t}-\sum_{t=0}^{D_{0}} {\Delta m}_{t}\rho^{-t} (20)$$

Note that the health state (h) disappears from equation (17), because the willingness to pay for changes in consumption and leisure is not influenced by health (although the utility derived from consumption and leisure is).

Equation (20) shows we can distinguish four potential causes of welfare changes:

1. A utility change due to a change in the total amount of time available for work and leisure (e_t_+l_t_), valued at the net wage rate
2. A utility change due to a change of the wage rate (w_t_).
3. A utility change due to a change in non-labour income (N_t_).
4. A utility change due to a change in medical expenditures ${(m}_{t}).$

The four causes lead to a new budget constraint, and therefore to another choice of leisure and working hours which optimizes utility in the new situation.

It is important to stress that it is the change in the total time that can be spent on leisure and work that (co)determines the new optimal utility level. The marginal utility of spending the (total) time available is equal for leisure and work. If working hours do not change and health deteriorates, leading to an increase of time needed for sleep and treatment, this leads to a reduction of the amount of leisure. Likewise, if an increase of time needed for sleep and treatment is fully compensated by a reduction in working hours, the quantity of leisure does not change. And if the reduction in working hours exceeds the increase of time needed for sleep and treatment, the amount of leisure increases. A health shock can thus lead to more, less or an equal amount of leisure. This depends on the trade-off between work and leisure in the new situation.

Equation (20) can be considered as a change in the willingness to pay for a life year in complete health. As equation (11) shows, the value of a QALY increases in consumption and leisure and decreases in health. So if consumption, leisure and health change, the average value of a QALY changes as well. Therefore, the willingness to pay for a life year in complete health will change if the amount of consumption and leisure changes. The question is whether, like for the value of a QALY, which for equity considerations is an average value over the whole population, such changes in the amount of consumption and leisure should also be averaged or could be calculated by wage rates specific for beneficiaries of an intervention. The latter implies that interventions for persons with lower wages yield lower gains. This both depends on practical issues, e.g. whether it is known upfront which persons will benefit from a specific intervention, as well as on equity considerations.

**Longevity**

In case of a positive change in life expectation from D_0_ to D_1_ the utility change for the patient corresponds to the last component of equation (8). This term can be further decomposed in a utility change due to extra life years in the new health state at the initial level of consumption and leisure and a utility change due to a change in the quantity of consumption and leisure in the extra life years:

$$\Delta U_{longevity, D_{1}>D_{0}}=\sum_{t=D_{0}+1}^{D_{1}} h_{t1}f(c_{t1},l_{t1})\rho^{-t}=\sum_{t=D_{0}+1}^{D_{1}} h_{t1}{f(c}_{t0},l_{t0})\rho^{-t} +\sum_{t=D_{0+1}}^{D_{1}} h_{t1}\Delta f(c_{t},l_{t})\rho^{-t} (21)$$

The social willingness to pay for the utility of an extra life year (the first term in equation (21)) is $V_{\mathrm{QALY}}$ (following from equation (14)). The willingness to pay for a change in the amount of consumption and leisure (the second term in equation (21)) is ${\Delta[(e_{t}+l}_{t}{)w}_{t}(1-i_{t})]+{\Delta N}_{t}-{\Delta m}_{t}$, as is shown in equation (20). Expressing the utility change in equation (21) in terms of willingness to pay and substituting these equations in equation (21) yields:

$$\mathrm{WTP}_{longevity, D_{1}>D_{0}}=\sum_{t=D_{0}+1}^{D_{1}} h_{t1}V_{\mathrm{QALY}}\rho^{-t} +\sum_{t=D_{0}+1}^{D_{1}} [\Delta\left[ \left( e_{t}+l_{t} \right)w_{t}(1-i_{t})]+{\Delta N}_{t} \right]\rho^{-t}-\sum_{t=D_{0}+1}^{D_{1}} {\Delta m}_{t}\rho^{-t} (22)$$

Note that the second term indicates that not the total loss or gain of consumption and leisure time during the added life years is taken into account, but the balance between a loss or gain in consumption and leisure time and a gain in non-labour income. This balance might be positive, zero or negative.

For negative changes in longevity (D_o_ >D_1_) the utility change for the patient corresponds to the last component of equation (9). This term cannot be further decomposed as it only contains the utility change due to a loss in life years. The willingness to pay for the loss in life years is:

$$\mathrm{WTP}_{longevity, D_{0}>D_{1}}=-\sum_{t=D_{1}+1}^{D_{0}} h_{t0}V_{\mathrm{QALY}}\rho^{-t} (23)$$

### Welfare change for the rest of society

To the welfare change of patients, the welfare change of the rest of society (non-patients) have to be added. These consist of transfers to and from the patient in the form of paid taxes, disability benefits, pensions and health cost covered by public insurance. The model assumes that the health state and longevity of individuals not affected by the intervention does not change. Depending on the way transfers are financed, net wages may change, which may have indirect effects on labour market participation. We assume however that there is no change of behaviour of the rest of society because of a change of transfers and thus include only the change of consumption caused by a change in transfers as a utility loss or gain for the rest of society. The change in consumption amounts to the change in non-labour income of the patient (N) plus a change in taxes paid by the patient induced by a change in working hours and by a change in the wage rate, through $e_{t}w_{t}i_{t}$. Thus the change in transfers from the rest of society to a patient in case of added life years is (adding a minus sign because we now take the perspective of the rest of society instead of the patients’ perspective):

$$\Delta transfers=-\left\{ \sum_{t=0}^{D_{1}} {[N}_{t1}-e_{t1}w_{t1}i_{t1}]\rho^{-t} -\sum_{t=0}^{D_{0}} {[N}_{t0}-e_{t0}w_{t0}i_{t0}]\rho^{-t} \right\} (24)$$

As the marginal willingness to pay for transfers is by definition 1, the change in welfare can be approximated by separating transfers in the original lifespan and the added life years:

$$\mathrm{WTP}_{transfers, D_{1}>D_{0}=}\sum_{t=0}^{D_{0}} {[N}_{t0}-e_{t0}w_{t0}i_{t0}]\rho^{-t}-\sum_{t=0}^{D_{0}} {[N}_{t1}-e_{t1}w_{t1}i_{t1}]\rho^{-t}-\sum_{t=D_{0}+1}^{D_{1}} {[N}_{t1}-e_{t1}w_{t1}i_{t1}]\rho^{-t}=\sum_{t=0}^{D_{0}} \Delta{[N}_{t}-e_{t}w_{t}i_{t}]\rho^{-t}-\sum_{t=D_{0}+1}^{D_{1}} {[N}_{t1}-e_{t1}w_{t}i_{t}]\rho^{-t} (25)$$

Equation (25) reflects the external costs or benefits of the intervention born by members of the rest of society. The first term denotes a change in transfers due to a change in health during the initial lifetime, for example a change in disability allowances, or a change in health costs covered by public insurance. The second term denotes the transfers in the added life years at the new level of consumption and leisure. Note that this term means that all transfers in the extra life years, computed at the initial level of consumption and leisure, are costs or benefits for the rest of society.

For negative changes in longevity (D_o_ >D_1_) equation (25) becomes:

$$\mathrm{WTP}_{transfers,Do >D1}=\sum_{t=0}^{D_{1}} {[N}_{t0}-e_{t0}w_{t0}i_{t0}]\rho^{-t}+\sum_{t=D_{1}+1}^{D_{0}} {[N}_{t0}-e_{t0}w_{t0}i_{t0}]\rho^{-t}-\sum_{t=0}^{D_{1}} {[N}_{t1}-e_{t1}w_{t1}i_{t1}]\rho^{-t} =-\sum_{t=0}^{D_{1}} \Delta{[N}_{t}-e_{t}w_{t}i_{t}]\rho^{-t}+\sum_{t=D_{1}+1}^{D_{0}} {[N}_{t0}-e_{t0}w_{t0}i_{t0}]\rho^{-t} (26)$$

### Total welfare change

Using equations (14), (20), (22) and (25), the total value of the welfare change in terms of marginal willingness to pay in case of additional life years can be calculated as:

$$\mathrm{WTP}_{\mathrm{total}}=\sum_{t=0}^{D_{0}} {\Delta h}_{t}V_{\mathrm{QALY}}\rho^{-t}+\sum_{t=0}^{D_{0}} \Delta\left[ \left( e_{t}+l_{t} \right)w_{t}(1-i_{t})+N_{t} \right]\rho^{-t}-\sum_{t=0}^{D_{0}} {\Delta m}_{t}\rho^{-t}-\sum_{t=0}^{D_{0}} \Delta{[N}_{t}-\left( e_{t}w_{t}i_{t} \right)]\rho^{-t}+\sum_{t=D_{0}+1}^{D_{1}} h_{t1}V_{\mathrm{QALY}}\rho^{-t}+\sum_{t=D_{0}+1}^{D_{1}} \Delta[\left[ \left( e_{t}+l_{t} \right)w_{t}(1-i_{t})]+N_{t} \right]\rho^{-t}-\sum_{t=D_{0}+1}^{D_{1}} {\Delta m}_{t}\rho^{-t}-\sum_{t=D_{0}+1}^{D_{1}} {[N}_{t1}-e_{t1}w_{t1}i_{t1}]\rho^{-t} (27)$$

In case of lost life years the welfare change in terms of marginal willingness to pay can be calculated using equations (15), (20), (23) and (26):

$$\mathrm{WTP}_{total=}\sum_{t=0}^{D_{1}} {\Delta h}_{t}V_{\mathrm{QALY}}\rho^{-t}+\sum_{t=0}^{D_{1}} \Delta\left[ e_{t}w_{t}+l_{t}w_{t}\left( 1-i_{t} \right)+N_{t} \right]\rho^{-t}-\sum_{t=0}^{D_{1}} {\Delta m}_{t}\rho^{-t}-\sum_{t=0}^{D_{1}} \Delta{[N}_{t}-e_{t}w_{t}i_{t}]\rho^{-t}- \sum_{t=D_{1}+1}^{D_{0}} h_{t0}V_{\mathrm{QALY}}\rho^{-t}+\sum_{t=D_{1}+1}^{D_{0}} {[N}_{t0}-e_{t0}w_{t0}i_{t0}]\rho^{-t} (28)$$

Note that the 6^th^ and the 7^th^ term of equation (27) are missing in equation (28), as there are no added life years. Lost life years only appear in the equation as the value of lost QALY’s, and a change in transfers: a loss of taxes that would have been paid by the patients in the lost life years and a gain in terms of a decline of transfers to the patients in the lost life years (like pensions and unrelated healthcare costs).

Balancing transfers, the total value of the welfare change in terms of marginal willingness to pay can, in case of additional life years (equation (27)), be calculated as:

$$\mathrm{WTP}_{\mathrm{total}}=\sum_{t=0}^{D_{0}} {\Delta h}_{t}V_{\mathrm{QALY}}\rho^{-t}+\sum_{t=D_{0}+1}^{D_{1}} h_{t1}V_{\mathrm{QALY}}\rho^{-t}+\sum_{t=0}^{D_{1}} \Delta\left[ e_{t}w_{t}+l_{t}w_{t}\left( 1-i_{t} \right) \right]\rho^{-t}+\sum_{t=D_{0}+1}^{D_{1}} e_{t1}w_{t1}i_{t1}\rho^{-t}-\sum_{t=0}^{D_{1}} {\Delta m}_{t}\rho^{-t}-\sum_{t=D_{0}+1}^{D_{1}} N_{t0}\rho^{-t} (29)$$

In case of lost life years the total welfare change adds up to:

$$\mathrm{WTP}_{total=}\sum_{t=0}^{D_{1}} {\Delta h}_{t}V_{\mathrm{QALY}}\rho^{-t}-\sum_{t=D_{1}+1}^{D_{0}} h_{t0}V_{\mathrm{QALY}}\rho^{-t}+\sum_{t=0}^{D_{1}} \Delta\left[ e_{t}w_{t}+l_{t}w_{t}\left( 1-i_{t} \right) \right]\rho^{-t}-\sum_{t=D_{1}+1}^{D_{0}} e_{t0}w_{t0}i_{t0}\rho^{-t}-\sum_{t=0}^{D_{1}} {\Delta m}_{t}\rho^{-t}+ \sum_{t=D_{1}+1}^{D_{0}} N_{t0}\rho^{-t} (30)$$

The total welfare change due to a change in health in equation (29) can be decomposed in four parts.:

1. The direct welfare change due to a change in health. The first term denotes the direct welfare change due to a change in the quality of consumption and leisure during the original life span. It is the health change multiplied by the value of a QALY. The second term is the willingness to pay for added life years. It is the quality of life in the new health state multiplied by the added life years.
2. The indirect welfare change due to a change in working and leisure time of the patient. It consists of the third term which denotes the change in the quantity of consumption and leisure during the original life span and in the added or lost life years. It also consists of the fourth term, which is the change in income taxes paid by the patient, due to a change in longevity.
3. Related medical costs: The fifth term is the change in medical costs due to the illness during the original life span and in the added life years. In case of lost life years it are the related medical costs until the new time of death.
4. A change in transfers to the patient due to a positive change in longevity. The last term denotes the transfers to the patient due to a change in longevity at the original level of consumption and leisure before the change in health. This term includes non-labour income like pensions and subsidies for unrelated medical costs. These transfers are not cancelled out because their counterpart is included in the value of a QALY.

From a societal perspective, most transfers are cancelled out, because they are a benefit for one but a cost for another. Not cancelled out is the change in taxes paid due to a change in working hours. The welfare change due to a change in working hours for the patient is related to the net wage, while the welfare change for society is related to the gross wage. So, from a societal perspective the total change in the value of production has to be taken into account. Furthermore, the costs of transfers of society to survivors (the last term in equations (29) and (30)) are not cancelled out. This is because the welfare change of these transfers for the patient is already captured in the willingness to pay for the extra life years (the second term in equation (29) and (30)) as they are included in the value of a QALY (see equation (11)). The opposite welfare change for the rest of society must thus be added.

### Implementation in cost-utility analysis

Equation (29) can be rewritten as a cost-effectiveness ratio. To be cost-effective the marginal willingness to pay for the intervention must be positive. Separating the QALY gain from the value of a QALY in the first two terms yields:

$${\mathrm{WTP}_{\mathrm{total}}=V}_{\mathrm{QALY}}\left[ \sum_{t=0}^{D_{0}} {\Delta h}_{t}\rho^{-t}+\sum_{t=D_{0}+1}^{D_{1}} h_{t1}\rho^{-t} \right]+\sum_{t=0}^{D_{1}} \Delta\left[ e_{t}w_{t}+l_{t}w_{t}\left( 1-i_{t} \right) \right]\rho^{-t}-\sum_{t=0}^{D_{1}} {\Delta m}_{t}\rho^{-t}-\sum_{t=D_{0}+1}^{D_{1}} {[N}_{t0}-e_{t1}w_{t1}i_{t1}]\rho^{-t}>0 (31)$$

Rewriting yields:

$$\frac{-\sum_{t=0}^{D_{1}} \Delta\left[ e_{t}w_{t}+l_{t}w_{t}\left( 1-i \right) \right]\rho^{-t}+\sum_{t=0}^{D_{1}} {\Delta m}_{t}\rho^{-t}+\sum_{t=D_{0}+1}^{D_{1}} {[N}_{t0}-e_{t0}w_{t0}i_{t0}]\rho^{-t}}{\sum_{t=0}^{D_{0}} {\Delta h}_{t}\rho^{-t}+\sum_{t=D_{0}+1}^{D_{1}} h_{t1}\rho^{-t}}<V_{\mathrm{QALY}} (32)$$

The numerator contains the cost of the intervention and the productivity changes. It also contains the cost of society of transfers to survivors. The denominator contains the QALY gain due to a higher quality of life and due to extra life years.

# References

1. Varian HR. *Microeconomic Analysis*. WW Norton & Company; 2004.
2. Borjas G. *Labor Economics*. McGraw-Hill/Irwin; 2013.
3. Hammitt JK. Admissible utility functions for health, longevity, and wealth: Integrating monetary and life-year measures. *J Risk Uncertain*. 2013;47(3):311-325. doi:10.1007/s11166-013-9178-4
